# Accelerated Aging in Major Depression: The Role of Nitro-Oxidative Stress

**DOI:** 10.1155/2013/230797

**Published:** 2013-11-14

**Authors:** Maria Luca, Antonina Luca, Carmela Calandra

**Affiliations:** ^1^Psychiatry Unit, Department of Medical and Surgery Specialties, University Hospital Policlinico-Vittorio Emanuele, Via S. Sofia 78, Catania, 95100 Sicily, Italy; ^2^Section of Neuroscience, Department of GF Ingrassia, University Hospital Policlinico-Vittorio Emanuele, Via S. Sofia 78, Catania, 95100 Sicily, Italy

## Abstract

Nitro-oxidative stress (NOS) plays a fundamental role in aging, as well as in the pathogenesis of neurodegenerative disorders, and major depression (MD). The latter is a very frequent psychiatric illness characterized by accelerated aging, neurodegeneration, high comorbidity with age-related disorders, and premature mortality; all of these conditions find an explanation in an altered redox homeostasis. If aging, neurodegeneration, and major depression share a common biological base in their pathophysiology, common therapeutic tools could be investigated for the prevention and treatment of these disorders. As an example, antidepressants have been demonstrated to present neuroprotective and anti-inflammatory properties and to stimulate neurogenesis. In parallel, antioxidants that stimulate the antioxidant defense systems and interact with the monoaminergic system show an antidepressant-like activity. Further research on this topic could lead, in the near future, to the expansion of the therapeutic possibilities for the treatment of NOS-related disorders.

## 1. Nitro-Oxidative Stress 

Reactive oxygen/nitrogen species (ROS/RNS) are by-products of cellular metabolism, primarily generated from mitochondria [1]. More specifically, ROS are reactive molecules derived from oxygen that can be free radicals (superoxide), hydroxyl radical (the most reactive and potentially cytotoxic species), or nonradicals (hydrogen peroxide). They can also be classified as ions (superoxide) and nonions (hydrogen peroxide). RNS, instead, are reactive species derived from nitrogen that can be classified as ions (peroxynitrite) or nonions (nitric oxide). ROS and RNS are involved in many physiological processes, such as cellular response to stress, modulation of autophagy, mitochondrial network, signaling, and apoptosis [2, 3]. However, being highly reactive species, they can lead to nitro-oxidative damage of proteins, lipids, DNA, and sugars, thus negatively affecting the cellular functioning [4, 5]. The potentially deleterious effects of ROS and RNS are neutralized by the endogenous antioxidative defense systems that include nonenzymatic and enzymatic antioxidants, such as glutathione, vitamin C, flavonoids, bilirubin, superoxide dismutase, catalases, and glutathione peroxidase [6, 7]. In addition, certain compounds are termed “upstream antioxidants,” since they prevent the formation of ROS/RNS (e.g., anti-inflammatory drugs, calcium antagonists). When the redox homeostasis (balance between oxidants-nitrosants production and elimination) fails, thus resulting in a preponderance of reactive species, “nitro-oxidative stress” (N and OS) occurs [8].

## 2. NOS and Aging

NOS plays a central role in aging. The “oxidative stress hypothesis” of aging is supported by some evidence: (a) the species life-span relates to antioxidant activity; (b) the enhanced expression of antioxidative enzymes increases longevity; (c) the free radical damage and the nitrosylation of proteins increase with age; (d) a reduced calorie intake decreases the production of ROS and increases life-span [9]. Hence, oxidative damage caused by ROS would contribute to the impaired physiological function, increased incidence of disease, and reduced life-span that characterize old age [10]. The progressive accumulation of oxidated, misfolded proteins is typical of the aging process and plays a central role in the pathogenesis of neurodegenerative disorders, which are in fact mostly considered as age-related illnesses [11, 12]. The brain seems to be particularly susceptible to NOS damage, particularly with aging, when the antioxidants defence mechanisms are less effective [13]. Some data highlight the importance of NOS stress in brain aging: (a) oxidative DNA damage, primarily that affecting mitochondrial DNA (mtDNA), increases in the aged brain [13–15]; (b) polyunsatured fatty acids are considerably susceptible to ROS and are depleted in the elderly, with consequences on cognition [13, 16]; (c) aging and age-related disorders have been related to inflammation, which stimulates the activation of microglia; the latter represents a defensive response, but it is also a source of free radicals and its prolonged activation leads to oxidative damage and neuronal cell death [13, 17]. The “mitochondrial free radical theory of aging,” assuming that ROS-related mtDNA damage leads to aging, has been questioned in light of recent evidence indicating that (a) mtDNA mutations may be generated by replication errors rather than by accumulated oxidative damage and (b) ROS mediate the stress response to age-related damage, being involved in signalling [18]. These new acquisitions, then, suggest a more complex role of oxidative stress in aging. As a matter of fact, ROS play a central role in the decline of mitochondrial respiratory function, as well as in the occurrence of mtDNA mutations and alterations of gene expression typical of aging [19]. Oxidative stress, through the alteration of epigenetic enzyme activity, DNA-methylation, and histone modifications, could contribute to the “aging epigenome”, that is linked to the deep impairment of the aged tissues [20]. Even RNS, through nitrosylation and nitration, play a role in signaling as well as in the modulation of aging. The activation of transcription factors due to age-related nitrosative stress seems to be an important cause of age-related diseases. For example, peroxynitrite leads to the activation of the nuclear factor kappa B, that has been demonstrated to be involved in the pathogenesis of neurodegenerative disorders and cancer [9, 21]. As a matter of fact, NOS is known to play a fundamental role not only in the pathogenesis of a considerable number of nonneurodegenerative disorders (such as diabetes [22], atherosclerosis [23], glaucoma [8], and psychiatric disorders [24]) but also and especially in the pathogenesis of neurodegenerative ones (e.g., Alzheimer's disease, amyotrophic lateral sclerosis, and multiple sclerosis [25–27]).

## 3. Accelerated Aging in Major Depression (MD): The Role of NOS Stress

Major depression (MD) is a very frequent psychiatric illness [28, 29] characterized by accelerated aging, neurodegeneration, high comorbidity with age-related disorders, and premature mortality [30, 31]. The clinical picture of MD, along with strictly affective symptoms, frequently include deficits in cognitive functions (e.g., impairment in attention, working memory, and executive function) [32, 33]. Sometimes, cognition is so deeply impaired that the differential diagnosis with dementia becomes necessary; in those cases, the term “depressive pseudodementia” is then used [34]. Brain functioning and neuronal plasticity are deeply alterated in MD, thus giving an explanation for depressive symptoms pertaining to the impairment in memory and concentration [35]. Anatomical alterations have been demonstrated in this disorder, such as volume reduction of critical cerebral areas (e.g., prefrontal cortex) [36], loss of neuronal cells [35, 37], and reduced cerebral blood flow [38]. Oxidative stress has been demonstrated to be involved in the occurrence of cognitive disturbances [39, 40]. In addition, increased levels of oxidative stress and/or antioxidant deficiencies represent risk factors for cognitive decline [41]. NOS stress seems to exert a role in the pathogenesis of MD and its associated conditions, such as accelerated aging, neurodegeneration, high comorbidity with age-related disorders, and increased mortality [30, 31]. MD is characterized by (a) less efficient antioxidant defence system [42]; (b) NOS; (c) inflammatory-neurodegenerative condition [31, 43]. The impaired antioxidant defence system exposes the body to the negative effects of ROS and to higher levels of oxidative stress, as demonstrated by the high levels of biomarkers of NOS stress in MD [44]. We previously mentioned the evidence linking NOS and aging; similar findings have been reported in MD. In fact, oxidative mtDNA damage has been demonstrated in this psychiatric disorder, which can even represent a manifestation of certain mitochondrial diseases [45–47]. In addition, even in MD there is a reduction in the amount of polyunsatured fatty acids, particularly in critical cerebral areas, such as the prefrontal cortex [48, 49]. High levels of antibodies against oxidized lipids have also been demonstrated [31]. Inflammation, which leads to an increased production of reactive species [43], is now universally recognized as a key point in the pathogenesis of MD [50]: the activation of the hypothalamic-pituitary-adrenal (HPA) axis, the activation of the microglia, and the production of proinflammatory cytokines and prostaglandins are mechanisms yet reported in the literature [51, 52]; in addition, the “sickness behaviour,” related to the production of proinflammatory cytokines, recalls the symptoms of depression [50]. The cytokines pathway appears to be involved in the mechanisms that can lead to the possible progression from depression to dementia [53]. Hence, NOS and inflammation share a role in the pathopsysiology of MD [31, 51] and could also offer an explanation of the frequent association between depression and neurodegeneration [31], which makes some scholars wonder if depression should be considered as a neurodegenerative disorder [44]. This new interpretation of MD acquires further credibility if the deep alterations in terms of neurogenesis, occurring in this disorder, are taken into consideration [54]. In the light of the previously reported data, it appears clear that MD shares similar biological alterations with aging and with age-related illnesses, neurodegenerative ones in particular. In fact, depression is often associated with cardiovascular disease, stroke, dementia, and Alzheimer [53, 55, 56], and it is characterized by anticipated gene expression changes (e.g., downregulation of the brain derived neurotrophic factor (BDNF)), which usually occurs in the aged brain [57]. The same genes involved in normal brain aging can be responsible for brain-related disorders; according to the model of “age-by-disease molecular interactions,” brain aging would stimulate the biological changes that, along with environmental factors and genetic variability, can favor the occurrence of age-related diseases [58]. The accelerated aging occurring in MD is also demonstrated by the fact that, in this psychiatric disorder, there is a shortening in the telomeres length, that represents a cumulative measure of stress [30, 59, 60]. Telomeres are DNA-protein complexes that cap the ends of linear DNA strands, thus protecting DNA from damage [30]; the cumulative exposure to NOS and inflammation is implicated in the shortening process [30], with the latter leading to premature cell death [30, 59, 61]. All these findings support the hypothesis that NOS plays a central role in the occurrence of accelerated aging in MD.

## 4. NOS in Aging, Neurodegeneration, and MD: Therapeutic Implications in Brief

NOS is deeply involved in aging, neurodegeneration, and major depression, which are conditions strictly linked to each other [9, 31, 55]. This evidence has not only a speculative value, but also therapeutic implications. In fact, if aging, neurodegeneration, and major depression share a common biological base in their pathophysiology, common therapeutic tools could be investigated for the prevention and treatment of these disorders. As an example, antidepressants have been demonstrated to present neuroprotective and anti-inflammatory properties [52, 62, 63] and to stimulate neurogenesis [62]. Chronic antidepressants drug treatments seem to favor the expression of BDNF, thus protecting neurons from the detrimental effects of stress [64]. The increase of BDNF, together with the reduction of microglia activation and oxidative stress, offers an explanation of the protective action of pre- and posttreatments with the antidepressant escitalopram against experimental ischemic neuronal damage [65]. In parallel, antioxidants, that stimulate the antioxidant defense systems and interact with the monoaminergic system, show an antidepressant-like activity [66–68] and are fundamental for a healthy neurophysiology [69]. For example, the beneficial effects of the dietary supplementation with omega-3 fatty acids on aging as well as on cardiovascular, neurological, and psychiatric disorders are yet known [69]. Moreover, the antidepressant profile of folic acid is partly due to its antioxidant properties [70].

## 5. Concluding Remarks

**Figure 1:**
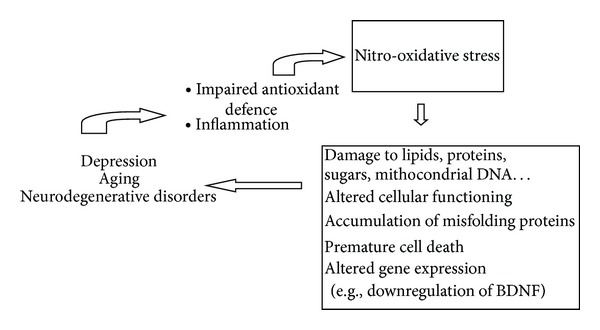
Inflammation and the impaired antioxid antioxidant defence expose the cell to the detrimental effects of nitro-oxidative stress, thus resulting in the deep molecular and functional alterations underlying depression, aging, and neurodegenerative disorders. BDNF: brain-derived neurotrophic factor.

Although the specific molecular mechanisms underlying aging, depression, and neurodegeneration are not deeply known, the evidence so far reported brings us to the following “biological cascade” (summarized in Figure 1): the imbalance between production and elimination of ROS/RNS (due to the impaired antioxidant defence, as well as to the inflammatory condition demonstrated in the three pathophysiological phenomena) exposes the cell to N and OS-related damages. The consequential alterations affect cell functioning, gene expression, proteins folding, and so forth and represent the “fertile ground” for aging-depression-neurodegenerative disorders. Since these alterations are shared by the three pathophysiological phenomena, their frequent association seems to find a biological explanation. Further research on this topic could lead, in the near future, to the expansion of the therapeutic possibilities for the treatment of N and OS-related disorders.

## References

[1] Thannickal VJ, Fanburg BL (2000). Reactive oxygen species in cell signaling. *The American Journal of Physiology—Lung Cellular and Molecular Physiology*.

[2] Bolisetty S, Jaimes EA (2013). Mitochondria and reactive oxygen species: physiology and pathophysiology. *International Journal of Molecular Sciences*.

[3] Baran CP, Zeigler MM, Tridandapani S, Marsh CB (2004). The role of ROS and RNS in regulating life and death of blood monocytes. *Current Pharmaceutical Design*.

[4] Gella A, Durany N (2009). Oxidative stress in Alzheimer disease. *Cell Adhesion and Migration*.

[5] Ogino K, Wang DH (2007). Biomarkers of oxidative/nitrosative stress: an approach to disease prevention. *Acta Medica Okayama*.

[6] Klandorf H, van Dyke K, Lushchak V (2012). Oxidative and nitrosative stresses: their role in health and disease in man and birds. *Oxidative Stress—Molecular Mechanisms and Biological Effects*.

[7] Khassaf M, McArdle A, Esanu C (2003). Effect of vitamin C supplements on antioxidant defence and stress proteins in human lymphocytes and skeletal muscle. *Journal of Physiology*.

[8] Fiedorowicz M, Grieb P, Gonzalez-Quevedo A (2012). Nitrooxidative stress and neurodegeneration. *Brain Damage—Bridging Between Basic Research and Clinics*.

[9] Ljubuncic P, Gochman E, Reznick AZ, Bondy S, Maiese K (2010). Nitrosative stress in aging—its importance and biological implications in NF-*κ*B signaling. *Aging and Age-Related Disorders*.

[10] Kregel KC, Zhang HJ (2007). An integrated view of oxidative stress in aging: basic mechanisms, functional effects, and pathological considerations. *The American Journal of Physiology—Regulatory Integrative and Comparative Physiology*.

[11] Nakamura T, Cho DH, Lipton SA (2012). Redox regulation of protein misfolding, mitochondrial dysfunction, synaptic damage, and cell death in neurodegenerative diseases. *Experimental Neurology*.

[12] Morawe T, Hiebel C, Kern A, Behl C (2012). Protein homeostasis, aging and Alzheimer’s disease. *Molecular Neurobiology*.

[13] Gemma C, Vila J, Bachstetter A, Bickford PC, Riddle DR (2007). Oxidative stress and the aging brain: from theory to prevention. *Brain Aging: Models, Methods, and Mechanisms*.

[14] Lu T, Pan Y, Kao SY (2004). Gene regulation and DNA damage in the ageing human brain. *Nature*.

[15] Corral-Debrinski M, Horton T, Lott MT, Shoffner JM, Beal MF, Wallace DC (1992). Mitochondrial DNA deletions in human brain: regional variability and increase with advanced age. *Nature Genetics*.

[16] Yehuda S, Rabinovitz S, Carasso RL, Mostofsky DI (2002). The role of polyunsaturated fatty acids in restoring the aging neuronal membrane. *Neurobiology of Aging*.

[17] Luo XG, Ding JQ, Chen SD (2010). Microglia in the aging brain: relevance to neurodegeneration. *Molecular Neurodegeneration*.

[18] Lagouge M, Larsson NG (2013). The role of mitochondrial DNA mutations and free radicals in disease and ageing. *Journal of Internal Medicine*.

[19] Lee HC, Wei YH (2007). Oxidative stress, mitochondrial DNA mutation, and apoptosis in aging. *Experimental Biology and Medicine*.

[20] Cencioni C, Spallotta F, Martelli F (2013). Oxidative stress and epigenetic regulation in ageing and age-related diseases. *International Journal of Molecular Sciences*.

[21] Mattson MP, Camandola S (2001). NF-*κ*B in neuronal plasticity and neurodegenerative disorders. *Journal of Clinical Investigation*.

[22] Calabrese V, Cornelius C, Leso V (2012). Oxidative stress, glutathione status, sirtuin and cellular stress response in type 2 diabetes. *Biochimica et Biophysica Acta*.

[23] Peluso I, Morabito G, Urban L, Ioannone F, Serafini M (2012). Oxidative stress in atherosclerosis development: the central role of LDL and oxidative burst. *Endocrine, Metabolic and Immune Disorders—Drug Targets*.

[24] Ng F, Berk M, Dean O, Bush AI (2008). Oxidative stress in psychiatric disorders: evidence base and therapeutic implications. *International Journal of Neuropsychopharmacology*.

[25] Seven A, Aslan M, Incir S, Altıntaş A (2013). Evaluation of oxidative and nitrosative stress in relapsing remitting multiple sclerosis: effect of corticosteroid therapy. *Folia Neuropathologica*.

[26] Butterfield DA, Castegna A, Drake J, Scapagnini G, Calabrese V (2002). Vitamin E and neurodegenerative disorders associated with oxidative stress. *Nutritional Neuroscience*.

[27] Mancuso C, Scapagnini G, Currò D (2007). Mitochondrial dysfunction, free radical generation and cellular stress response in neurodegenerative disorders. *Frontiers in Bioscience*.

[28] Luca M, Prossimo G, Messina V, Luca A, Romeo S, Calandra C (2013). Epidemiology and treatment of mood disorders in a day hospital setting from 1996 to 2007: an Italian study. *Neuropsychiatric Disease and Treatment*.

[29] Bromet E, Andrade LH, Hwang I (2011). Cross-national epidemiology of DSM-IV major depressive episode. *BMC Medicine*.

[30] Wolkowitz OM, Mellon SH, Epel ES (2011). Leukocyte telomere length in major depression: correlations with chronicity, inflammation and oxidative stress—preliminary findings. *PLoS ONE*.

[31] Maes M, Mihaylova I, Kubera M, Uytterhoeven M, Vrydags N, Bosmans E (2010). Increased plasma peroxides and serum oxidized low density lipoprotein antibodies in major depression: markers that further explain the higher incidence of neurodegeneration and coronary artery disease. *Journal of Affective Disorders*.

[32] Ravnkilde B, Videbech P, Clemmensen K, Egander A, Rasmussen NA, Rosenberg R (2002). Cognitive deficits in major depression. *Scandinavian Journal of Psychology*.

[33] Marazziti D, Consoli G, Picchetti M, Carlini M, Faravelli L (2010). Cognitive impairment in major depression. *European Journal of Pharmacology*.

[34] Richly P, Abdulhamid P, Bustin J (2012). Depressive pseudodementia. Differential diagnosis or meeting point?. *Vertex*.

[35] Michel TM, Pülschen D, Thome J (2012). The role of oxidative stress in depressive disorders. *Current Pharmaceutical Design*.

[36] Ozalay O, Calli C, Kitis O (2013). The relationship between the anterior corpus callosum size and prefrontal cortex volume in drug-free depressed patients. *Journal of Affective Disorders*.

[37] Rajkowska G (2002). Cell pathology in mood disorders. *Seminars in Clinical Neuropsychiatry*.

[38] Orosz A, Jann K, Federspiel A (2012). Reduced cerebral blood flow within the default-mode network and within total gray matter in major depression. *Brain Connect*.

[39] Johnson AW, Jaaro-Peled H, Shahani N (2013). Cognitive and motivational deficits together with prefrontal oxidative stress in a mouse model for neuropsychiatric illness. *Proceedings of the National Academy of Sciences of the United States of America*.

[40] Fukui K, Omoi NO, Hayasaka T (2002). Cognitive impairment of rats caused by oxidative stress and aging, and its prevention by vitamin E. *Annals of the New York Academy of Sciences*.

[41] Berr C, Balansard B, Arnaud J, Roussel AM, Alpérovitch A (2000). Cognitive decline is associated with systemic oxidative stress: the EVA study. Etude du Vieillissement Artériel. *Journal of the American Geriatrics Society*.

[42] Maes M, de Vos N, Pioli R (2000). Lower serum vitamin E concentrations in major depression. Another marker of lowered antioxidant defenses in that illness. *Journal of Affective Disorders*.

[43] Maes M, Yirmyia R, Noraberg J (2009). The inflammatory & neurodegenerative (I&ND) hypothesis of depression: leads for future research and new drug developments in depression. *Metabolic Brain Disease*.

[44] Maes M, Galecki P, Chang YS, Berk M (2011). A review on the oxidative and nitrosative stress (O&NS) pathways in major depression and their possible contribution to the (neuro)degenerative processes in that illness. *Progress in Neuro-Psychopharmacology and Biological Psychiatry*.

[45] Tobe EH (2013). Mitochondrial dysfunction, oxidative stress, and major depressive disorder. *Neuropsychiatric Disease and Treatment*.

[46] Jou SH, Chiu NY, Liu CS (2009). Mitochondrial dysfunction and psychiatric disorders. *Chang Gung Medical Journal*.

[47] Anglin RE, Garside SL, Tarnopolsky MA, Mazurek MF, Rosebush PI (2012). The psychiatric manifestations of mitochondrial disorders: a case and review of the literature. *Journal of Clinical Psychiatry*.

[48] McNamara RK, Liu Y (2011). Reduced expression of fatty acid biosynthesis genes in the prefrontal cortex of patients with major depressive disorder. *Journal of Affective Disorders*.

[49] Hamazaki K, Hamazaki T, Inadera H (2012). Fatty acid composition in the postmortem amygdala of patients with schizophrenia, bipolar disorder, and major depressive disorder. *Journal of Psychiatric Research*.

[50] Raedler TJ (2011). Inflammatory mechanisms in major depressive disorder. *Current Opinion in Psychiatry*.

[51] Nunes SO, Vargas HO, Prado E (2013). The shared role of oxidative stress and inflammation in major depressive disorder and nicotine dependence. *Neuroscience and Biobehavioral Reviews*.

[52] Luca M, Luca A, Celia A, Calandra C Prostaglandins pathway as a possible biological link between cancer and major depression.

[53] Leonard BE (2007). Inflammation, depression and dementia: are they connected?. *Neurochemical Research*.

[54] Bewernick BH, Schlaepfer TE (2013). Chronic depression as a model disease for cerebral aging. *Dialogues in Clinical Neuroscience*.

[55] Wolkowitz OM, Reus VI, Mellon SH (2011). Of sound mind and body: depression, disease, and accelerated aging. *Dialogues in Clinical Neuroscience*.

[56] Maes M, Mihaylova I, Kubera M, Uytterhoeven M, Vrydags N, Bosmans E (2009). Increased 8-hydroxy-deoxyguanosine, a marker of oxidative damage to DNA, in major depression and myalgic encephalomyelitis/chronic fatigue syndrome. *Neuroendocrinology Letters*.

[57] Douillard-Guilloux G, Guilloux JP, Lewis DA, Sibille E (2013). Anticipated brain molecular aging in major depression. *The American Journal of Geriatric Psychiatry*.

[58] Sibille E (2013). Molecular aging of the brain, neuroplasticity, and vulnerability to depression and other brain-related disorders. *Dialogues in Clinical Neuroscience*.

[59] Kinser PA, Lyon DE (2013). Major depressive disorder and measures of cellular aging: an integrative review. *Nursing Research and Practice*.

[60] Wikgren M, Maripuu M, Karlsson T (2012). Short telomeres in depression and the general population are associated with a hypocortisolemic state. *Biological Psychiatry*.

[61] Blackburn EH (2000). Telomere states and cell fates. *Nature*.

[62] Young LT (2002). Neuroprotective effects of antidepressant and mood stabilizing drugs. *Journal of Psychiatry and Neuroscience*.

[63] Hashioka S, McGeer PL, Monji A, Kanba S (2009). Anti-inflammatory effects of antidepressants: possibilities for preventives against alzheimer’s disease. *Central Nervous System Agents in Medicinal Chemistry*.

[64] Nibuya M, Morinobu S, Duman RS (1995). Regulation of BDNF and trkB mRNA in rat brain by chronic electroconvulsive seizure and antidepressant drug treatments. *Journal of Neuroscience*.

[65] Lee CH, Park JH, Yoo KY (2011). Pre- and post-treatments with escitalopram protect against experimental ischemic neuronal damage via regulation of BDNF expression and oxidative stress. *Experimental Neurology*.

[66] Smaga I, Pomierny B, Krzyżanowska W (2012). N-acetylcysteine possesses antidepressant-like activity through reduction of oxidative stress: behavioral and biochemical analyses in rats. *Progress in Neuro-Psychopharmacology and Biological Psychiatry*.

[67] Jesse CR, Wilhelm EA, Bortolatto CF, Nogueira CW (2010). Evidence for the involvement of the serotonergic 5-HT2A/C and 5-HT3 receptors in the antidepressant-like effect caused by oral administration of bis selenide in mice. *Progress in Neuro-Psychopharmacology and Biological Psychiatry*.

[68] Gerzson MFB, Victoria FN, Radatz CS (2012). In vitro antioxidant activity and in vivo antidepressant-like effect of *α*-(phenylselanyl) acetophenone in mice. *Pharmacology Biochemistry and Behavior*.

[69] Mazza M, Pomponi M, Janiri L, Bria P, Mazza S (2007). Omega-3 fatty acids and antioxidants in neurological and psychiatric diseases: an overview. *Progress in Neuro-Psychopharmacology and Biological Psychiatry*.

[70] Budni J, Zomkowski AD, Engel D (2013). Folic acid prevents depressive-like behavior and hippocampal antioxidant imbalance induced by restraint stress in mice. *Experimental Neurology*.

